# Metformin inhibits small intestinal neuroendocrine tumor growth in vivo

**DOI:** 10.1186/s12885-026-16418-z

**Published:** 2026-06-25

**Authors:** Fredrik Axling, Samuel Backman, Per Hellman, Olov Norlén, Elham Barazeghi, Peter Stålberg

**Affiliations:** Department of Surgical Sciences, Rudbeck Laboratory, Uppsala University, Uppsala University Hospital, Uppsala, SE-751 85 Sweden

**Keywords:** Neuroendocrine tumors, metformin, SI-NETs, RNA-seq, miRNA-seq

## Abstract

**Background:**

Small intestinal neuroendocrine tumors (SI-NETs) are slow-growing but highly metastatic, with most patients presenting metastases at diagnosis. Radical surgery remains the only potential curative option when feasible. Consequently, there is a critical need for novel therapeutic strategies that can limit tumor progression and enable more personalized treatment approaches in combination with current clinical practices. In this study, we evaluated the impact of metformin on SI-NET cell growth in vivo, characterized the associated microRNA expression profile, and identified potential driver genes modulated by metformin treatment.

**Methods:**

A total of 22 SI-NET xenograft mouse models were established using CNDT2.5 and GOT1 cells. Mice were treated with metformin (2.56 mg/mL in drinking water) or water as control for 4 weeks. To explore the molecular impact of metformin, both small-RNA and total-RNA sequencing were performed on the dissected xenograft tumors. Proliferation and apoptosis were further evaluated by immunohistochemistry.

**Results:**

In vivo treatment of SI-NET cells with metformin led to a reduction in tumor size in both CNDT2.5 and GOT1 xenograft models. Our sequencing analyses identified seven altered microRNAs and 1,776 differentially expressed genes in metformin-treated tumors compared to controls. To uncover potential driver genes in SI-NETs affected by metformin, we compared the differentially expressed genes from GOT1 xenograft model with those identified by comparing single-cell RNA profile of enterochromaffin cells to SI-NETs. This novel approach revealed a set of significantly regulated genes, including those involved in tumor proliferation, apoptosis, and metastasis, as well as genes related to voltage-gated calcium channels and signal transduction.

**Conclusions:**

Our novel findings support further investigation of metformin as a potential therapeutic agent in clinical trials for SI-NET patients, and suggest that identified miRNAs should be assessed as potential predictive biomarkers for metformin treatment. This study highlights novel candidate driver genes affected by metformin, which are associated with key cellular processes and enterochromaffin cell’s function, offering insights into the underlying mechanisms in SI-NETs.

**Supplementary Information:**

The online version contains supplementary material available at 10.1186/s12885-026-16418-z.

## Introduction

Small intestinal neuroendocrine tumors (SI-NETs) are relatively rare and slow-growing neoplasms. However, these are the most common tumors of the small intestine and exhibit a high metastatic potential, with most patients presenting metastases at the time of diagnosis. Around 20–30% of the tumors secrete serotonin and other vasoactive peptides, which leads to carcinoid syndrome [1]. Radical surgery remains the only potential curative treatment for these patients when feasible [2]. Therefore, there is a critical need for novel therapeutic options to reduce the tumor growth and improve individualized treatment approaches in combination with current strategies.

Metformin, a medication widely prescribed for the treatment of type 2 diabetes, has demonstrated anticancer activity in several preclinical studies including neuroendocrine tumors [3, 4]. Previously, we showed that metformin treatment repressed the cell viability of SI-NET cells and inhibited the growth of the cell spheroids in vitro [5]. Recent clinical trials have also reported promising outcomes when metformin was combined with the commonly used treatments, such as lanreotide or everolimous [6, 7]. However, understanding the detailed mechanisms underlying the anti-proliferative effect of metformin in cancer cells requires further investigations.

MicroRNAs (miRNAs) are small, non-coding RNAs that post-transcriptionally regulate the gene expression, and have been shown to be reliable biomarkers for predicting treatment response in several cancers [8, 9]. Accumulating evidence indicates that metformin exerts anticancer effects through multiple pathways including modulation of miRNAs, although these effects vary across different cancer types. In human pancreatic cancer cells, metformin inhibits cell proliferation and tumor growth by modulating miR-150 and miR-7 [10]. In breast cancer, metformin reduces cell growth and invasiveness through upregulation of miR-200 [11]. Similarly, in melanoma, metformin suppresses cell growth and motility by increasing the expression of miR-192 and miR-584 [12]. Given the potential of miRNAs as prognostic biomarkers for treatment and that miRNA modulation is involved in mechanism of action of metformin, we investigated the miRNA expression profiles of the metformin treated SI-NET cells.

In this study, we investigated the effect of metformin treatment on the growth of SI-NET cells in vivo. We also elucidated the miRNA signature in response to metformin treatment and revealed potential driver genes affected by metformin in SI-NETs.

## Methods

### Animal model

Female, NMRI-nude mice (5–6 weeks old) were purchased from Charles River, and three mice per cage were housed at the Rudbeck laboratory animal facility (Uppsala University, Sweden) in individually ventilated cages. All experiments and animal maintenance were approved by and performed according to the regulations and guidelines of the Uppsala Animal Ethics Committee (ID number 5.8.18–07081/2019). All animal experiments were performed according to the guidelines of ARRIVE (Animal Research: Reporting of In Vivo Experiments).

### Establishment of tumor xenografts

CNDT2.5 adhesive cells [13, 14] developed from a liver metastasis in a patient diagnosed with primary ileal SI-NET, were kindly provided by Dr. Lee Ellis, MD, Anderson Cancer Center, Houston, TX, USA, and used at cell passages 10–30 in this study. GOT1 adhesive cells [15, 16] were a kind gift from Dr. Ola Nilsson, Sahlgrenska Cancer Center, University of Gothenburg, Sweden. Both cell lines expressed the neuroendocrine cell marker synaptophysin. CNDT2.5 cells were washed twice, counted, resuspended in PBS at 5 × 10^6^, and mixed 1:1 (vol/vol) with Matrigel (Corning) in a total volume of 100 µL. The cells were injected subcutaneously in the hind flank of the mice (*n* = 19), which produced palpable xenograft tumors after 7 days. One mouse was excluded from the study due to complications after cell inoculation. 18 mice were randomly assigned into vehicle control (water) and metformin treatment groups (*n* = 9 per group). At day0 of treatment, the mean tumor volume (mm3) ± standard deviation for CNDT2.5 control was 113.3 ± 27.08 and metformin = 75.22 ± 25.44. The metformin treatment group (*n* = 9) received 2.56 mg/mL of metformin dissolved in drinking water for 4 weeks, and their water was changed twice a week. After 28 days of treatment, the mice were euthanized by using carbon dioxide (CO2) inhalation, and the xenograft tumors were dissected. 2 × 10^7^ GOT1 cells were prepared and inoculated in the hind flank of mice (*n* = 12), as mentioned above. After 43 days, only four mice carried a palpable xenograft tumor, which were randomly assigned into two groups: vehicle control (*n* = 2) and metformin treatment (*n* = 2). At day0 of treatment, the mean tumor volume (mm3) ± standard deviation for GOT1 control was 108.78 ± 22.94 and metformin = 91.45 ± 22.92. Xenograft tumors were dissected after 30 days of treatment. Twice a week, tumor growth was monitored by caliper measurement, and the mice were weighed. Tumor size was calculated using an ellipsoid volume formula (length × width × depth × π/6). The dissected tumors were divided into two parts. One part was snap-frozen in liquid nitrogen and stored at -70^o^ C, and the other part was paraffin-embedded.

### Immunohistochemistry

Paraffin-embedded tumor sections were deparaffinized with xylene and rehydrated through descending alcohol concentrations and distilled water. 3% hydrogen peroxide was used to block the background staining. Then, the sections were heated in citrate buffer pH 6.0 and treated with the proper normal serum and the rabbit monoclonal anti-synaptophysin antibody (ab32127, Abcam), the mouse monoclonal anti-Ki-67 antibody clone MIB-1 (M7240, Dako), or the rabbit polyclonal anti-caspase-3 active/cleaved form antibody (AB3623, Merck). After incubation with the proper secondary antibody and Avidin-Biotin Complex, diaminobenzidine (DAB) was used for visualization. A qualitative scoring system was used to estimate the proportion of positively stained cells for Ki-67 and the active form of caspase-3. Positive cells were counted in three randomly selected fields and categorized into two scoring ranges: 10–49% or 50–95%. CNDT2.5 and GOT1 cells were fixed in formalin and incubated for 20 min with ice-cold 70% ethanol. Then the slides were stained for synaptophysin, as described above.

### Extraction of nucleic acids, library preparation and sequencing

Frozen CNDT2.5 and GOT1 xenograft tumor sections were cut using a cryostat, and total RNA was isolated using the RNeasy Mini Kit (Qiagen) according to the manufacturer’s instructions. Isolated RNA underwent DNase I treatment with TURBO DNA-free (Ambion). RNA quality was evaluated for microRNA-sequencing using TapeStation RNA ScreenTape Analysis (Agilent), and miRNA concentration was determined using a Qubit microRNA assay (ThermoFisher Scientific). The QIAseq miRNA library kit (Qiagen) was used to prepare a library from 10 ng of total RNA. NextSeq500/550 (Illumina Inc.) was utilized to do 75 cycles of single-read sequencing to produce at least 10 million reads per sample. RNA quality and concentration for RNA-sequencing were evaluated using the TapeStation RNA ScreenTape Analysis (Agilent). The 2100 Bioanalyzer system (Agilent) was used to determine the RNA integrity number (RIN). 1 µg of total RNA was prepared utilizing the TruSeq stranded total RNA library preparation kit, with ribosomal depletion using RiboZero Gold (Illumina Inc.), followed by 150 cycles of paired-end sequencing on the NovaSeq X Plus system (Illumina Inc.), producing at least 80 million reads per sample.

### Bioinformatics analysis

MicroRNA-sequencing reads were quality controlled with a Q score of 30 by trimming and adapter removal using cutadapt [17]. UMI-tools [18] were used for extracting Unique Molecular Identifiers (UMI-tags) and follow-up read deduplication. Bowtie [19] was used for aligning against the GRCh38 reference genome with the following settings: “-n 1 -l 30 --norc --best --strata -m 1”. FeatureCounts [20] was used for counting microRNAs aligned to the reference genome matching sequences present in miRbase [21]. DESeq2 [22] was used for differential gene expression with statistical comparison. RNA-sequencing reads were quality controlled with a Q score of 30 by read trimming and adapter removal using Trim Galore [23]. Gene expression was quantified using salmon quant [24] with 24 bootstraps and correction for GC-bias, sequence-bias, and validated mappings against the GRCh38.110 transcriptome with genome decoys [25]. Results from salmon quantification were loaded into DESeq2 for differential gene expression and statistical comparison using BioMart [26] and Tximeta [27]. Prediction for miRNA-mRNA interactions was performed with starBase/ENCORI [28] with the following thresholds: CLIP-Data > = 5, Degradome-Data > = 0, pan-Cancer > = 5, and programNum > = 2. The STRING Database [29] was used to generate interaction networks between differentially expressed genes. Network visualization was performed in Cytoscape [30], and clustering was carried out with MCODE algorithm [31] to identify and resolve complex clusters. Functional enrichment analysis was performed using the STRING Database and g: Profiler [32] focusing on Gene Ontology (GO) terms (Cellular Component, Biological Process, Molecular Function), STRING clusters, Reactome Pathways, WikiPathways, KEGG Pathways, UniProt Keywords, DISEASES, Monarch Phenotype, and TISSUES.

### Statistics

R-statistics 4.31 was used for all statistical comparisons. For water intake, mouse weight, xenograft tumor volume, and CD44 expression between metformin treatment and control groups, Mann–Whitney U test was used to assess statistical differences. A p-value ≤ 0.05 was considered significantly different. RNA and microRNA expression differences between control and metformin treatment were assessed using a Wald test with p-value adjustment (q-value) for multiple testing using the Benjamini-Hochberg method, and a q-value ≤ 0.05 was deemed significant. For single-cell expression validation between SI-NET cells and enterochromaffin cells (EC), the Mann-Whitney U test was used with Bonferroni correction for multiple comparisons, with an adjusted p-value < 0.05 as significant. In the STRING database enrichment analysis, terms with a false discovery rate (FDR) < 0.05 were considered significant, whereas in the g: Profiler enrichment analysis, terms with a g: SCS threshold < 0.05 were deemed significant.

## Results

### Metformin inhibited tumor growth of SI-NET cells in vivo

**Fig. 1:**
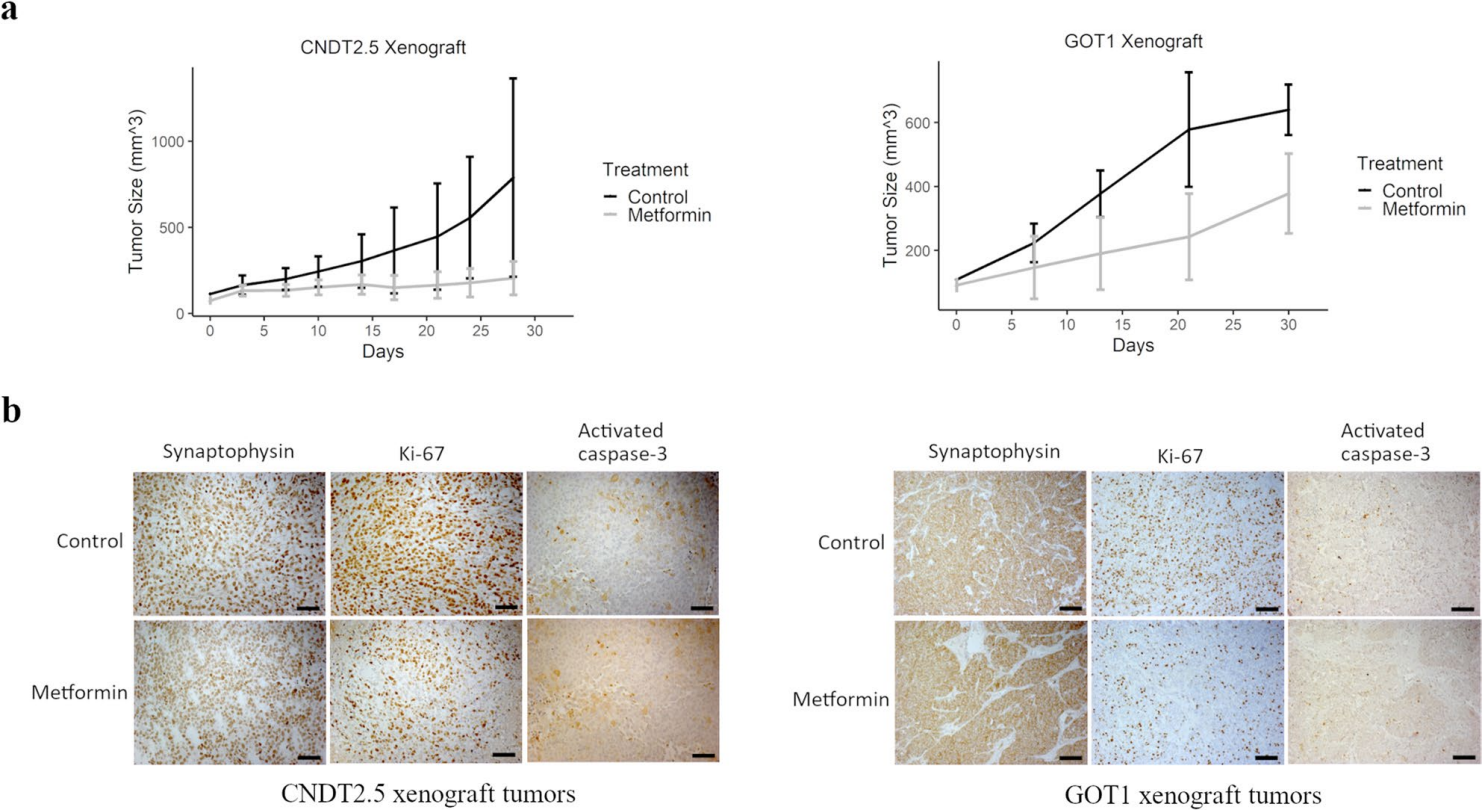
Metformin treatment in vivo. **a** NMRI-nude female mice, carrying xenograft tumors of CNDT2.5 (*n* = 18) or GOT1 (*n* = 4), were treated with either vehicle control (water) or metformin (2.56 mg/mL) for four weeks. A smaller tumor size was observed in the metformin treatment groups (CNDT2.5, day28, *p* = 0.0077). The mean tumor volume (mm^3^) ± standard deviation (SD) are presented. Day28, CNDT2.5 control = 789.09 ± 575.66, metformin = 204.99 ± 97.45 and day30, GOT1 control = 640.05 ± 79.19, metformin = 377.9 ± 124.68 (**b**) Representative results from immunohistochemical analysis of synaptophysin, Ki-67, and activated caspase-3 in tumors dissected from the control and metformin treated groups. Scale bar 100 μm

In order to investigate the effect of metformin treatment on the growth of SI-NETs in vivo, we used the SI-NET xenograft mouse model, as described before [5]. The metformin treatment groups received 2.56 mg/mL of metformin dissolved in drinking water for 4 weeks. Water intake by mice was not affected by metformin addition to the water. According to a previous study [33], metformin plasma concentrations in mice receiving metformin at 1–5 mg/mL in drinking water were consistent with values reported in diabetic patients. Furthermore, in line with previous studies [34, 35], the mice exhibited no side effects, and there was no significant variation in mouse weight observed between the control and treatment groups throughout the course of treatment (data not shown). After 30 days of treatment, the mice were euthanized, and the xenograft tumors were dissected. Additional file 1: Figure S1a and b show the tumors excised from the control and metformin treatment groups, and Figure S1c shows the tumor sizes at the time of dissection. Although the tumors showed variation in growth rate, the largest tumor sizes were obtained in the control group, and the metformin group had significantly smaller tumor sizes in CNDT2.5 xenograft tumors (day28, *p* = 0.0077) (Fig. 1a). Consistently, the tumor weight was lower in the metformin group than the controls for the CNDT2.5 and GOT1 xenograft tumors (Additional file 1: Figure S1d). Although the number of GOT1 xenograft tumors is very small, we observed a trend of reduction in the size of the tumors treated with metformin (Fig. 1a). All the tumors (*n* = 22) stained positively for the neuroendocrine cell marker synaptophysin, and consistent with our previous in vitro report [5], the expression of Ki-67 was reduced in the tumor tissues after metformin treatment compared with the control group (Additional file 1: Figure S1e), while no effect on activation of caspase-3 was detected. Representative staining results are shown in Fig. 1b.

### miRNA and gene expression profiling in response to metformin treatment in SI-NETs in vivo

**Fig. 2:**
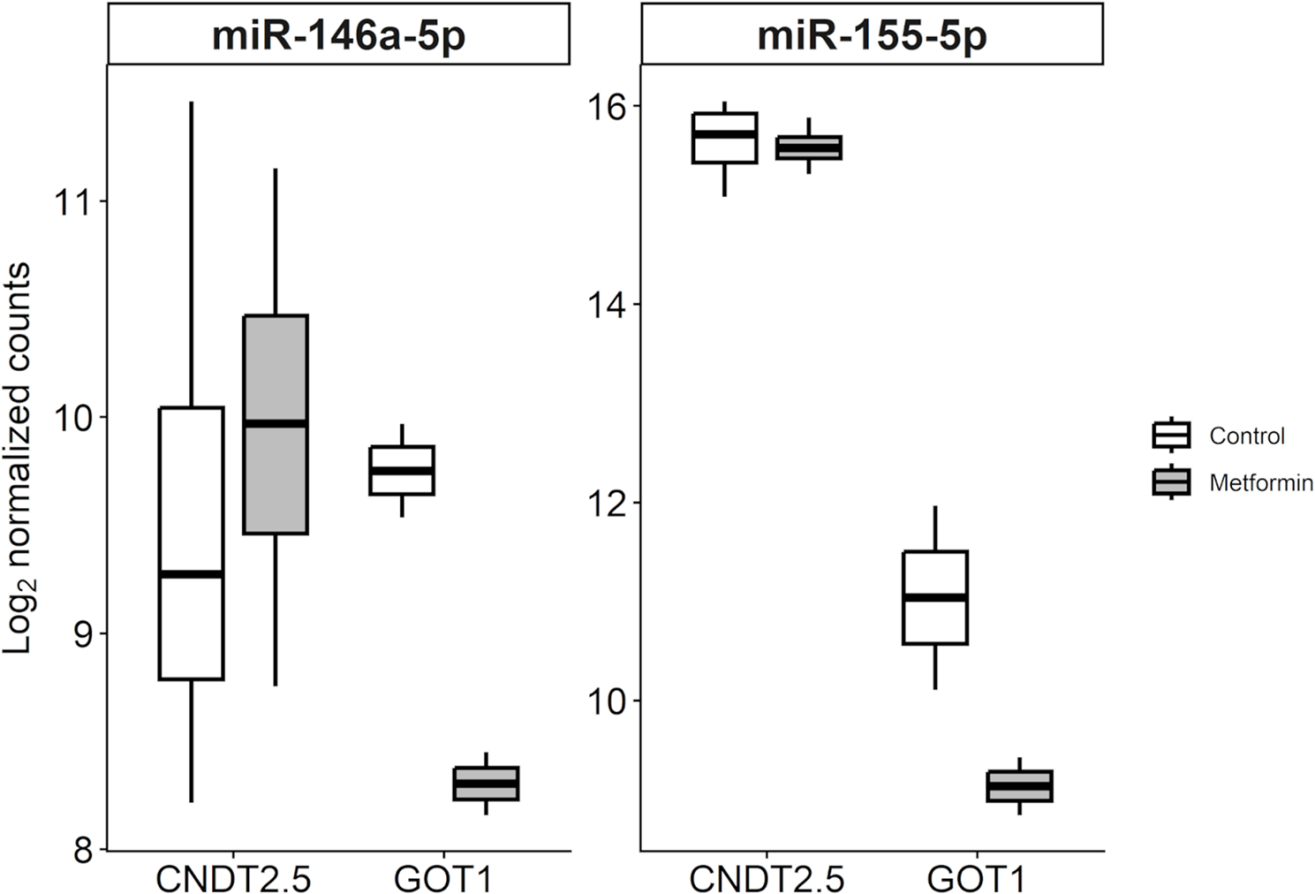
Expression levels of miR-146a-5p and miR-155-5p in CNDT2.5 (*n* = 8) and GOT1 (*n* = 4) xenograft tumors following metformin treatment compared to control. Presented as log_2_-transformed normalized counts

To search for potential biomarkers for predicting metformin treatment outcome, eight of the CNDT2.5 and four of the GOT1 xenograft tumors were subjected to small RNA sequencing. In GOT1 xenograft tumors, seven miRNAs showed lower expression in the metformin-treated group compared with the controls (Additional file 2: Table S1, q ≤ 0.05). Two of these miRNAs, miR-146a and miR-155, whose expression levels in GOT1 xenografts are shown in Fig. 2, have previously been reported to be inhibited by metformin in prostate and breast cancers, respectively [36, 37]. In CNDT2.5 xenograft tumors, no clear differences in miRNA expression were observed between treated and control groups (Fig. 2). To further validate our miRNA data, we analyzed a previously generated miRNA array profile of SI-NETs (*n* = 15) [38]. Both miR-146a and miR-155 were expressed at higher levels in these tumors compared with the immuno-microdissected normal EC cells. These two miRNAs were also found expressed in another miRNA array study in SI-NETs [39]. Overall, these findings suggest their potential as biomarkers.

**Fig. 3:**
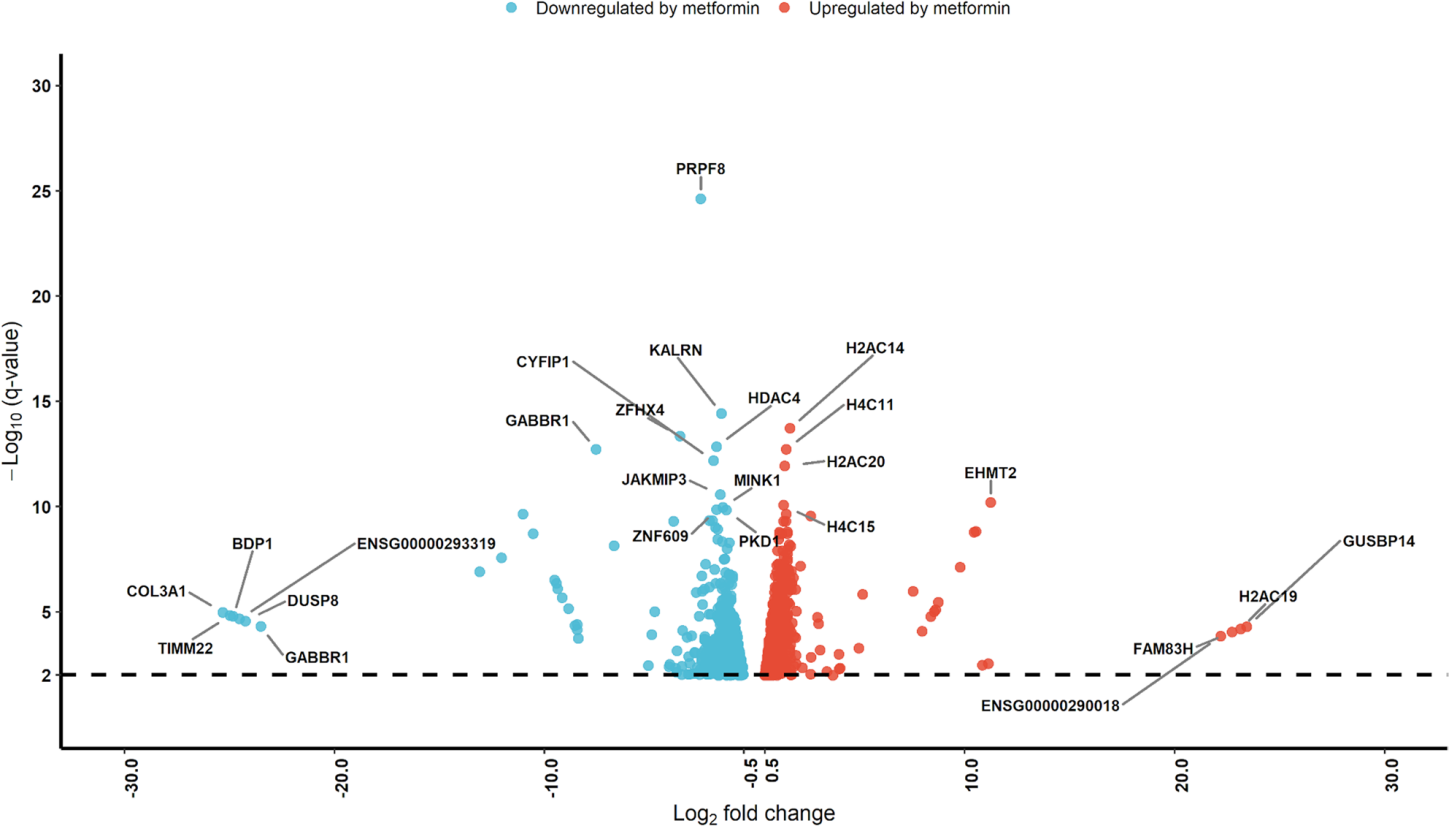
Volcano plot showing the top 15 DEGs and genes with log_2_ fold change greater than 20 or less than − 20 among genes identified as differentially expressed (*n* = 1776) in GOT1 xenografts. Dotted line indicates the q-value threshold of 0.01. Cut-off for log_2_ fold change is 0.5

To explore putative target genes of the miRNAs, we performed total RNA sequencing of the GOT1 xenograft tumors. This allowed the identification of 1776 genes that differed in expression between metformin-treated and control tumors using a threshold of q ≤ 0.01 (Fig. 3). Among these, 716 genes showed lower expression and 1060 showed higher expression by metformin treatment (Additional file 3: Table S2). Prediction of miRNA-mRNA interactions between the six (one was not identified by ENCORI/starBase) miRNAs and the genes differing in expression identified 118 interaction pairs, including 76 targets with higher expression and 42 with lower expression (Additional file 1: Figure S2 and Additional file 4: Table S3). Functional enrichment analysis of the 118 putative targets indicated associations with multiple metabolic-related pathways and cadherin binding proteins involved in cell adhesion (Additional file 1: Figure S3).

Functional interaction analysis of the 1776 genes using the STRING database suggested associations with protein networks such as NADH dehydrogenase and cytochrome c oxidase (Additional file 1: Figure S4 and Additional file 5: Table S4). To further support that the observed expression alterations may reflect a biological effect of metformin treatment, and in line with previous studies [40, 41], we observed lower CD44 expression, a common cell surface marker of the cancer stem cells, in metformin-treated xenografts compared to controls. (Additional file 1: Figure S5).

### Identification of potential driver genes modulated by metformin in SI-NETs

To further explore pathways potentially affected by metformin treatment, particularly those involving SI-NET driver genes, we used a recently generated single-cell RNA-seq dataset by us [42]. This dataset compares patient-derived EC cells with SI-NETs and identified 457 differentially expressed genes (DEGs). By comparing these 457 DEGs with those showing altered expression following metformin treatment, we identified 78 overlapping genes (q ≤ 0.05). Among these, 65 genes showed higher expression in metformin-treated tumors, while downregulated in SI-NETs compared to the normal EC cells. Moreover, we found 13 overexpressed genes in SI-NETs that showed lower expression by metformin treatment (Additional file 6: Table S5). Functional analysis of these overlapping genes highlighted 20 candidate genes of interest associated with cell signaling, cell adhesion and DNA repair (Table 1).

**Table 1:** Gene ontology of 20 selected dysregulated genes modulated by metformin

Symbol	Description	Molecular function	Biological process
Upregulated genes inhibited by metformin			
CACNA1B	calcium voltage-gated channel subunit alpha1 B	calcium ion binding	signaling and transmebrane transport
CACNA1D	calcium voltage-gated channel subunit alpha1 D	monoatomic ion channel activity	signaling and transmebrane transport
SNED1	sushi, nidogen and EGF like domains 1	calcium ion binding	cell adhesion
TRPS1	transcriptional repressor GATA binding 1	DNA-binding transcription factor activity	regulation of transcription and neuron differentiation
MYT1L	myelin transcription factor 1 like	DNA-binding transcription factor activity	regulation of transcription
QKI	QKI, KH domain containing RNA binding	nucleic acid binding	oncogenic MAPK signaling
BAIAP2	BAR/IMD domain containing adaptor protein 2	protein and SH3 domain binding	signaling by Rho GTPases
FMN2	formin 2	actin binding	cell migration and DNA repair
Downregulated genes induced by metformin			
S100A6	S100 calcium binding protein A6	calcium ion binding	signal transduction
CLIC1	chloride intracellular channel 1	chloride channel activity	signal transduction and chloride transport
MIF	macrophage migration inhibitory factor	signaling receptor binding	signaling and apoptosis
RGS2	regulator of G protein signaling 2	GTPase activator activity	signal transduction
FABP5	fatty acid binding protein 5	lipid binding	signaling and lipid metabolism
TFF3	trefoil factor 3	protein binding	signal transduction
TP53I3	tumor protein p53 inducible protein 3	oxidoreductase activity	Transcriptional Regulation by TP53
CLDN7	claudin 7	structural molecule activity	cell adhesion
POLD4	DNA polymerase delta 4, accessory subunit	DNA-directed DNA polymerase activity	DNA repair
POLR2L	RNA polymerase II, I and III subunit L	DNA binding	DNA-templated transcription
JUNB	JunB proto-oncogene	DNA-binding transcription factor activity	cell differentiation
ELOB	elongin B	translation elongation factor activity	translational elongation

## Discussion

Despite significant efforts, treatment options to effectively suppress tumor growth and metastasis remain limited for patients with SI-NETs. Metformin has emerged as a promising therapeutic agent in cancer treatment, including pancreatic NETs [4], leading to the initiation of several clinical trials [6, 7]. Our previous in vitro findings showed an anti-proliferative response in SI-NET cells by metformin treatment [5]. In this study, we showed that metformin treatment of CNDT2.5 and GOT1 xenografts was associated with reduced tumor size in nude mice and lower proliferation, as reflected by a reduced Ki-67 index in the dissected tumors. Our findings suggest the use of metformin as an anti-cancer drug alone or in combination with current treatment strategies. Notably, GOT1 xenografts were established with a success rate of only 33% after 43 days. This low success rate is primarily due to the markedly slower growth rate of GOT1 cells compared with more rapidly proliferating cell lines such as CNDT2.5, as GOT1 cells typically require culture expansion every two to three weeks. While this slow proliferation closely mimics the growth nature of SI-NETs, it poses a major challenge in the efficient development of xenograft models [43].

Given the role of miRNAs in key cancer hallmarks, and evidence from several studies [44, 45] indicating that metformin’s anti-tumor effects are mediated through alterations in miRNA expression, we investigated the miRNA profile of xenograft tumor tissues treated with metformin compared to the controls. In CNDT2.5 xenografts, no clear differences in miRNA expression were observed between groups, likely due to high intra-group variability, as reflected by the wide range of tumor sizes in the control group, despite an overall reduction in tumor size following treatment. Additionally, the effect of metformin on miRNA expression in CNDT2.5 cells may be too subtle to detect with our current analysis, given the small sample size of only eight CNDT2.5 xenografts. In contrast, seven miRNAs showed lower expression by metformin treatment of GOT1 xenograft tumors. Notably, among these, miR-1 and miR-146a were previously found highly expressed in primary tumors and metastases, respectively [46]. Indeed, miR-146a has been shown to regulate multiple target genes and pathways, playing diverse roles in different cancer types. In particular, its anti-apoptotic function has been demonstrated in gastric cancer and hepatocellular carcinoma, where it exerts its effects by targeting SMAD4 [47, 48]. Interestingly, reduced expression of SMAD4, located on chromosome 18, has been previously reported in SI-NETs [49]. miR-155 has also been previously reported to be upregulated in breast cancer, where it is related to increased invasiveness and metastatic potential. Furthermore, inhibition of miR-155 was found to reduce cell proliferation through activation of AMPK and suppressing TNFα signaling [50]. Both miR-146a and miR-155 have previously been reported to be suppressed by metformin [36, 37], and a previous miRNA array study [38] found them to be more highly expressed in SI-NETs compare with EC cells. These observations suggest that these two miRNAs may represent potential biomarkers. However, their value as predictive biomarkers for metformin treatment in SI-NETs requires further investigation and validation.

To better understand the molecular basis for the inhibitory effect of metformin, we analyzed gene expression in the GOT1 xenograft tumors, leading to the identification of 1776 DEGs. Among the genes highly downregulated by metformin, Collagen type III alpha 1 chain (COL3A1), an extracellular matrix protein expressed by cancer-associated fibroblasts, has been associated with malignant phenotypes in several cancers [51, 52]. Future research will be of interest to investigate whether metformin modulates the tumor microenvironment of SI-NETs through suppression of COL3A1 expression. A combined target prediction and pathway analysis for the altered miRNAs indicated association with metabolic pathways and cadherin-binding proteins. No Gene Ontology terms or pathways directly related to tumorigenesis were apparent. Consistent with previous studies [53, 54], our analysis also suggested involvement of protein networks related to metabolism, including NADH dehydrogenase and cytochrome c oxidase, among the genes with altered expression.

SI-NETs arise from EC cells, which constitute less than 1% of the total intestinal epithelial cells. Consequently, identifying driver genes in SI-NETs has been hampered by the lack of availability of appropriate normal tissue. To address this, we previously generated an expression dataset comparing normal EC cells with SI-NETs using single-cell RNA analysis [42]. In the current study, we compared genes with altered expression in GOT1 xenograft model with the DEGs identified in the single-cell analysis. This novel comparison enabled us to identify potential candidate driver genes in SI-NETs that are modulated by metformin treatment and revealed a number of overlapping genes, some of which have previously been implied that are associated with tumor proliferation, apoptosis as well as invasive growth and metastasis, including QKI, RGS2, CLDN7 and JUNB [55–58]. Of great interest is the observation that several of the dysregulated genes are involved in voltage-gated calcium channels and signal transduction. As demonstrated previously [59], EC cells rely on channel activity, specific receptors and signal transduction pathways to detect relevant stimuli within the gut and transmit this information to nervous system.

Our study has several limitations. First, due to the limited sample size, particularly for the GOT1 xenografts, caution is warranted when interpreting and generalizing the findings. Second, the single-cell RNA dataset was generated from only two patients, which may limit the broader applicability of the results. Third, the identified miRNAs and potential candidates require further experimental validation.

## Conclusions

Our findings suggest that metformin warrants further investigation as a potential therapeutic agent in clinical trials for patients with SI-NETs. Additionally, further analysis in a larger cohort is needed to evaluate the identified miRNAs as predictive biomarkers for metformin treatment. In this study, we identified novel candidate and potential driver genes affected by metformin, which are associated with various cellular processes and EC cell function. Investigating these genes may provide deeper insights into the mechanisms underlying metformin’s effects in these tumors.

## Supplementary Information

Supplementary Material 1. Figure S1. Metformin treatment of xenograft tumors. NMRI-nude female mice carrying (a) CNDT2.5 (n = 18) or (b) GOT1 (n = 4) xenograft tumors were treated with either vehicle control (water) or metformin (2.56 mg/mL). The xenograft tumors were dissected after four weeks of treatment. (c) Xenograft tumor sizes (mm3) at the time of dissection are presented. (d) Xenograft tumor weight is presented as means ± SEM. Tumor weights in the two groups of CNDT2.5 xenografts were compared using Mann–Whitney U test (*, p<0.05). (e) Immunohistochemical analysis of Ki-67 in dissected tumors from the control and metformin treated groups. Scale bar 100 µm. Figure S2. miRNA-mRNA interaction plot. Interaction network for the six miRNAs showing lower expression in metformin treated xenografts and the DEGs identified in the comparison of metformin treated vs control GOT1 xenografts. Figure S3. Gene ontology (GO) enrichment analysis of the 118 putative targets of the six identified miRNAs. The black line indicates p-value threshold. Figure S4. Gene expression clusters in GOT1 xenograft tumors based on a subset of 1776 DEGs using a threshold of q ≤ 0.01. The genes were clustered using a minimum interaction score of 0.9 and requiring at least one connected neighbor. Next, a weighted scoring algorithm was implemented to isolate specific clusters with a score of ≥ 12000. To assess their functional importance, each cluster was investigated using functional enrichment analysis (FDR < 0.05). Four sub-clusters was detected; (a) NADH dehydrogenase and cytochrome c oxidase cluster, (b) proteasome cluster, (c) spliceosome cluster, and (d) nucleosome cluster. Figure S5. CD44 expression in GOT1 xenograft tumors (p = 0.061).

Supplementary Material 2. Table S1. miRNA in GOT1 xenograft tumors (Excel 10 kb).

Supplementary Material 3. Table S2. DEGs in GOT1 xenograft tumors. (Excel 209 kb).

Supplementary Material 4. Table S3. miRNA-mRNA interactions. (Excel 26 kb).

Supplementary Material 5. Table S4. Gene expression clusters in GOT1 xenograft tumors. (Excel 105 kb).

Supplementary Material 6. Table S5. Dysregulated genes in SI-NETs affected by metformin. (Excel 14 kb).

## Data Availability

The datasets generated and/or analyzed during the present study are available from the corresponding authors on reasonable request.

## Abbreviations

SI-NETs: small intestinal neuroendocrine tumors
miRNA: micro RNA
NMRI: Naval Medical Research Institute
ARRIVE: Animal Research: Reporting of In Vivo Experiments
CO2: carbon dioxide
DAB: diaminobenzidine
mRNA: messenger RNA
FDR: false discovery rate
PBS: Phosphate Buffered Saline
DEG: differentially expressed gene
GO: gene ontology
ENCORI: Encyclopedia of RNA Interactomes
STRING: Search Tool for the Retrieval of Interacting Genes/Proteins
NADH: Nicotinamide adenine dinucleotide
EC: enterochromaffin
SMAD4: Mothers against decapentaplegic homolog 4
AMPK: Adenosine Monophosphate (AMP)-Activated Protein Kinase
TNFα: Tumor Necrosis Factor alpha
QKI: Quaking homolog, KH domain RNA binding
RGS2: Regulator of G-protein Signaling 2
CLDN7: Claudin-7
