# Supplementary figures and images for: Metformin inhibits small intestinal neuroendocrine tumor growth in vivo
Source: BMC Cancer. 2026 Jun 25;26:772. doi: 10.1186/s12885-026-16418-z (PMC13295228; doi:10.1186/s12885-026-16418-z)

Figure S1.

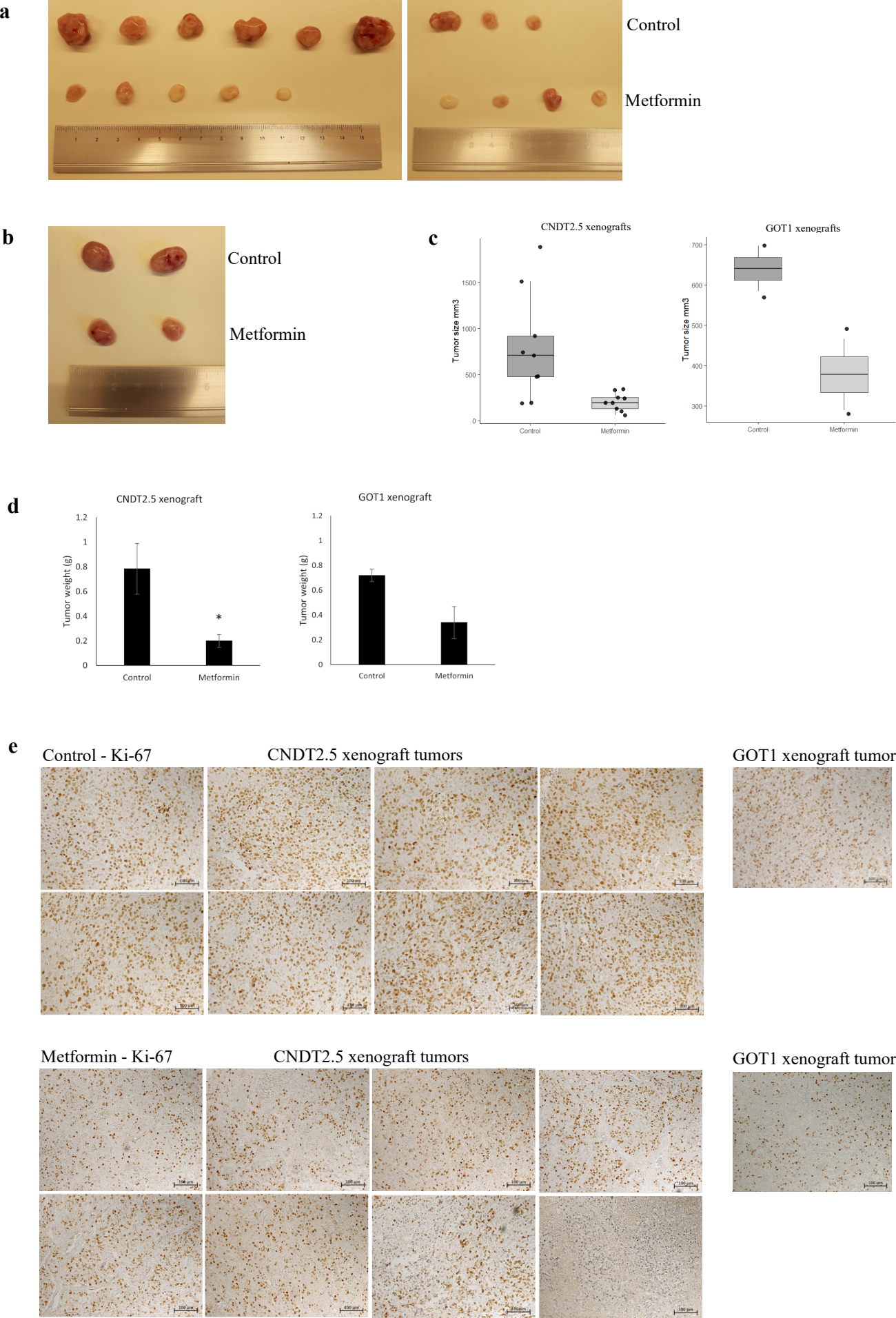

Figure S2.

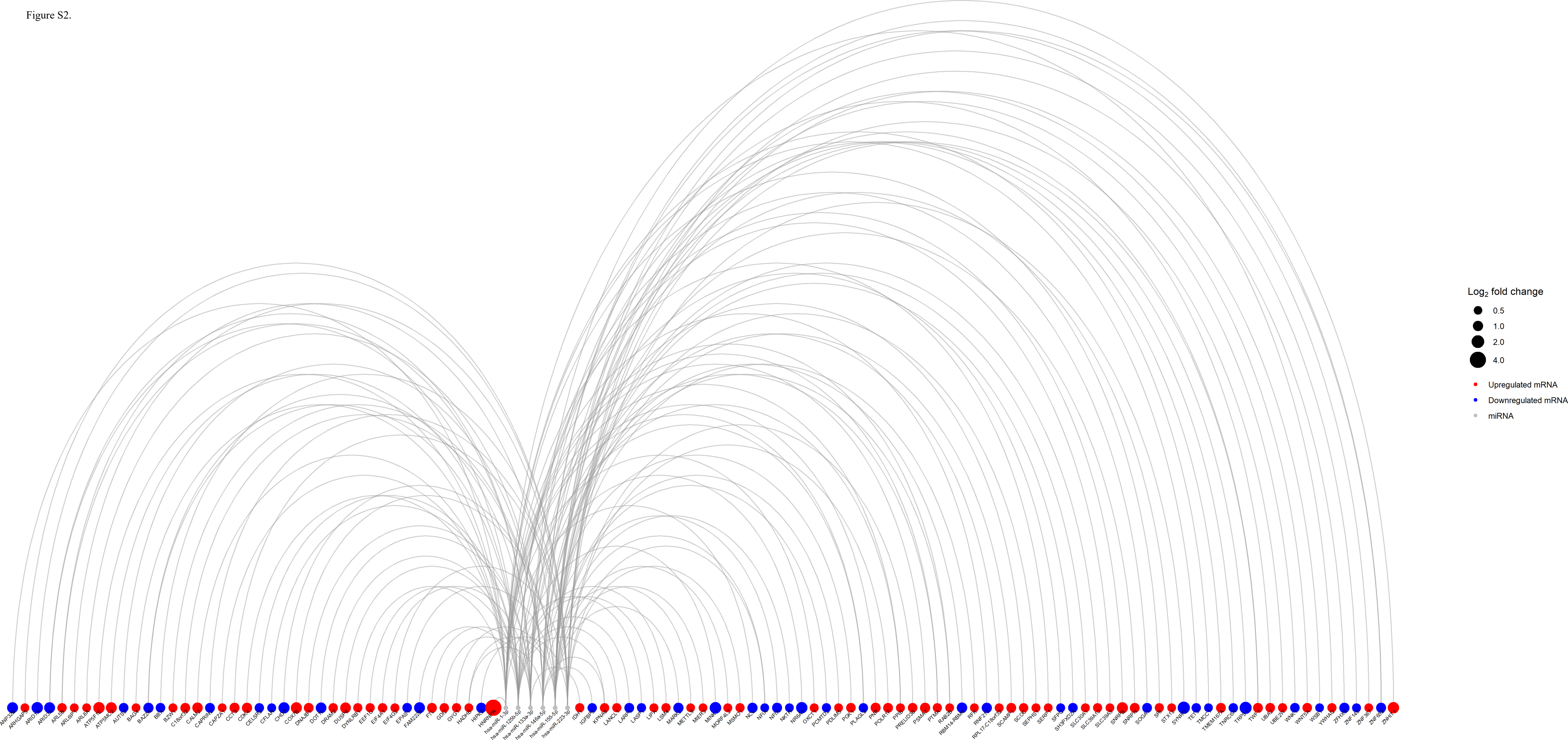

Figure S3.

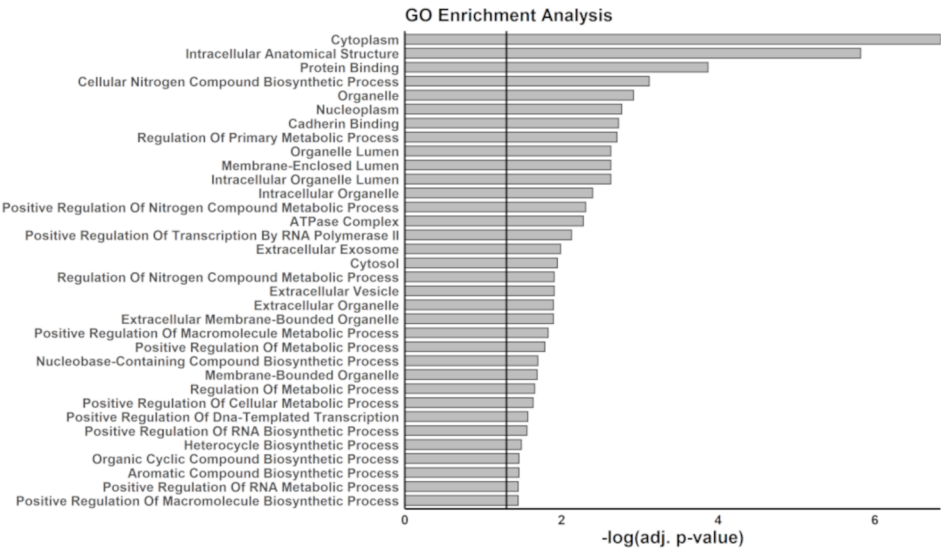

Figure S4.

**a**

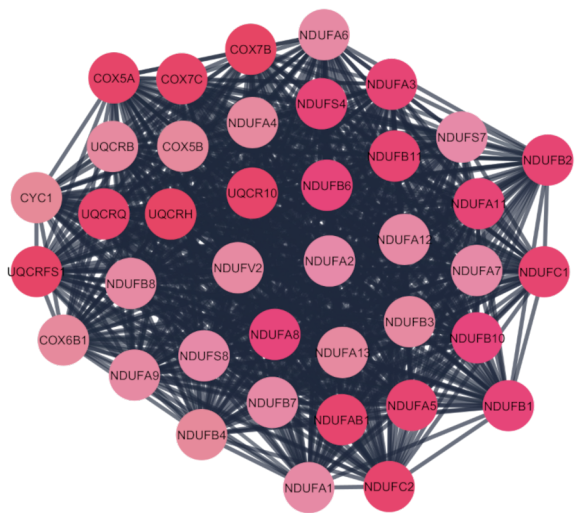

**b**

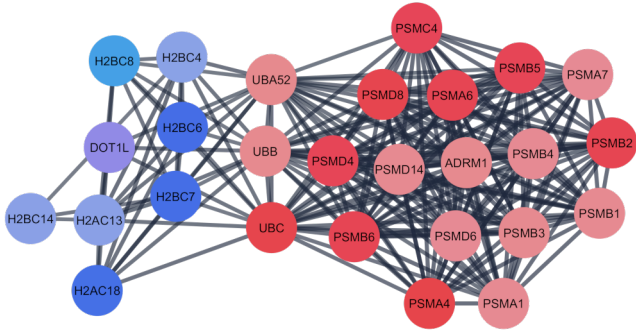

**c**

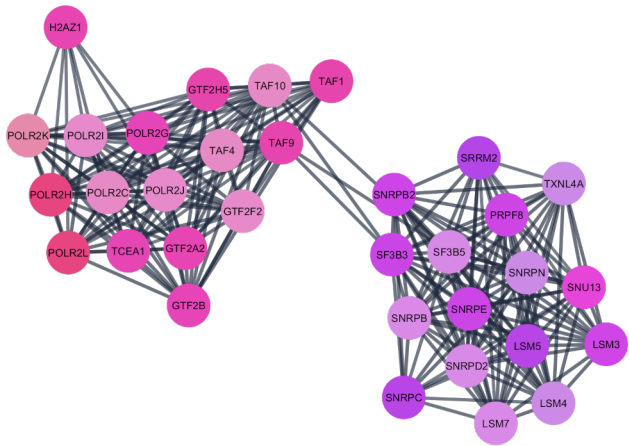

**d**

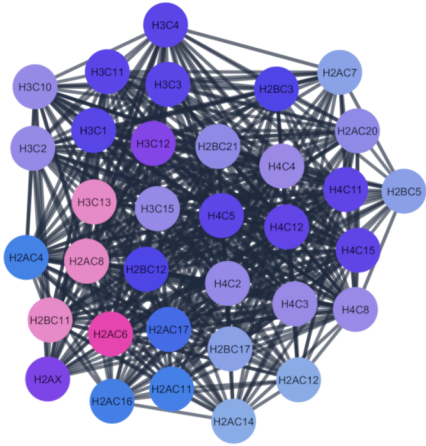

Figure S5.

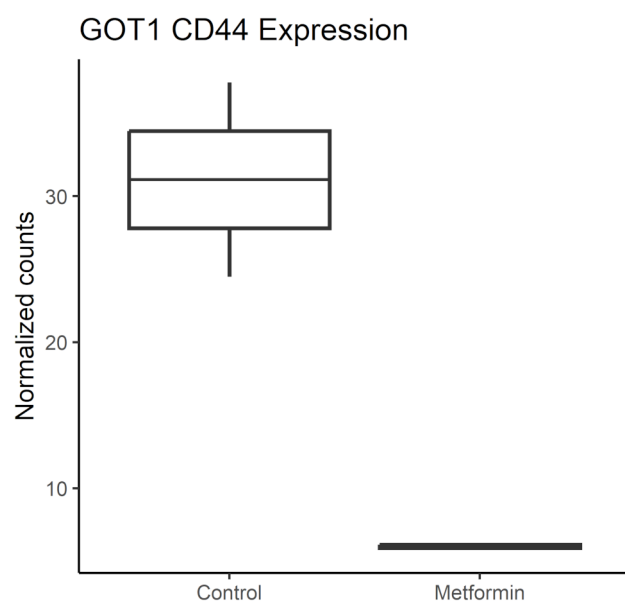

Supplement: Supplementary file 1 — Supplementary Material 1. Figure S1. Metformin treatment of xenograft tumors. NMRI-nude female mice carrying (a) CNDT2.5 (n = 18) or (b) GOT1 (n = 4) xenograft tumors were treated with either vehicle control (water) or metformin (2.56 mg/mL). The xenograft tumors were dissected after four weeks of treatment. (c) Xenograft tumor sizes (mm3) at the time of dissection are presented. (d) Xenograft tumor weight is presented as means ± SEM. Tumor weights in the two groups of CNDT2.5 xenografts were compared using Mann–Whitney U test (*, p<0.05). (e) Immunohistochemical analysis of Ki-67 in dissected tumors from the control and metformin treated groups. Scale bar 100 µm. Figure S2. miRNA-mRNA interaction plot. Interaction network for the six miRNAs showing lower expression in metformin treated xenografts and the DEGs identified in the comparison of metformin treated vs control GOT1 xenografts. Figure S3. Gene ontology (GO) enrichment analysis of the 118 putative targets of the six identified miRNAs. The black line indicates p-value threshold. Figure S4. Gene expression clusters in GOT1 xenograft tumors based on a subset of 1776 DEGs using a threshold of q ≤ 0.01. The genes were clustered using a minimum interaction score of 0.9 and requiring at least one connected neighbor. Next, a weighted scoring algorithm was implemented to isolate specific clusters with a score of ≥ 12000. To assess their functional importance, each cluster was investigated using functional enrichment analysis (FDR < 0.05). Four sub-clusters was detected; (a) NADH dehydrogenase and cytochrome c oxidase cluster, (b) proteasome cluster, (c) spliceosome cluster, and (d) nucleosome cluster. Figure S5. CD44 expression in GOT1 xenograft tumors (p = 0.061). [file 12885_2026_16418_MOESM1_ESM.pdf]
